# Opportunities and Challenges of Generic Pre-Exposure Prophylaxis Drugs for HIV

**DOI:** 10.1017/jme.2022.33

**Published:** 2022

**Authors:** Jeromie Ballreich, Timothy Levengood, Rena M. Conti

**Keywords:** Drug Policy, PrEP, HIV, Drug Pricing

## Abstract

Antiretroviral pre-exposure prophylaxis (PrEP) is protective against HIV. Low utilization rates amongst HIV vulnerable populations are due in part to the high cost of PrEP. Generic PrEP offers the potential to improve health at significantly reduced costs. In this study, we examine early utilization patterns and prices for generic PrEP. We discuss the opportunities and challenges for generic PrEP to improve health among HIV vulnerable populations.

## Background

Antiretroviral Pre-Exposure Prophylaxis (PrEP) is a medication used to reduce the risk of acquiring HIV. Studies have shown it to be protective against HIV, with an estimated 99% risk reduction from contracting HIV by having sex and a 74% risk reduction of contracting HIV through intravenous drug use.^1^ Despite the availability of this effective option, PrEP uptake amongst HIV vulnerable individuals in the U.S. remains low, with around 23% uptake across an estimated 1.2 million HIV vulnerable individuals.^2^ Racial disparities in PrEP access are also well documented, with black and Latino men and black women far less likely to be taking PrEP compared to white peers with similar HIV-risk.^3^ Policymakers recognize the importance of increased PrEP uptake and have explicitly targeted a 50% uptake of PrEP in the U.S. by 2030 as part of the national initiative, Ending the HIV Epidemic: A Plan for America.^4^

The reasons for low PrEP uptake in the U.S. are multi-factorial. Pinto et al. (2018) conducted a review of literature identifying barriers to PrEP uptake.^5^ Key barriers include cognitive barriers, such as understanding HIV risk; provider-centric barriers, including the “purview paradox” where patients who may benefit from PrEP are seen by primary care providers unfamiliar with PrEP; and health system barriers, such as lack of communication, prevalent homophobia, transphobia, and HIV-stigma, along with other access barriers. One of the major barriers driving low uptake in the U.S. is the high cost of PrEP.

Until recently, only two medications have been U.S. Food and Drug Administration (FDA) approved for the use of PrEP: the combination drug of emtricitabine and tenofovir disoproxil fumarate and the combination drug of emtricitabine and tenofovir alafenamide fumarate, both drugs marketed by Gilead Sciences as Truvada and Descovy, respectively.^6^ The first PrEP medication, the combination drug of emtricitabine and tenofovir disoproxil fumarate, was FDA approved in August 2004 for treatment of HIV (it was approved for prevention of HIV in 2012).^7^ For nearly 16 years, the company benefited from exclusive market protections on the branded drug, and then in October 2020 a generic was FDA approved for sale. During its time on the market without generic competition, the combination drug of emtricitabine and tenofovir disoproxil fumarate enjoyed significant price premiums with a list price of $1842 and estimated net price of $867 for a 30-day supply of the commonly recommended 200-300 mg dose in 2020.^8^

In this study, we examined recent price patterns for branded and generic PrEP in the U.S. We also examined early patterns of utilization of branded and generic PrEP in both the national U.S. market and across state Medicaid programs.

Previous research supports the contention that the advent of generic entry and competition in prescription drug markets benefits patients and other payers.^9^ Generic drug entry and competition results in significant price concessions in comparison to the brand.^10^ When the first generic product PrEP entered the U.S. market with six months of exclusivity, it was priced 10-15% lower than the brand, and recent generic PrEP prices across multiple manufacturers are 90% lower than branded PrEP.^11^ Despite the lower price for generic PrEP, we do not know the rates of generic PrEP utilization in the U.S. market. In theory, lower prices may result in similar levels of drug use or expanded use compared to that observed when only the brand was available for purchase. For policymakers, the lower prices of generic PrEP compared to the brand may help the U.S. reach its 2030 goal of 50% PrEP uptake among HIV vulnerable populations by improving the cost effectiveness of access efforts.^12^

In this study, we examined recent price patterns for branded and generic PrEP in the U.S. We also examined early patterns of utilization of branded and generic PrEP in both the national U.S. market and across state Medicaid programs.

## Methods

### Drug Sample

Our study identified branded and generic PrEP using the Drugs@FDA database.^13^ This database provided initial dates of FDA approval and provided the sample of drugs to evaluate the utilization and price patterns. Using the drug product and molecular names, we identified the National Drug Codes (NDCs) for branded and generic PrEP using the National Average of Drug Acquisition Cost (NADAC) database.^14^ The NADAC database is maintained by the Centers for Medicare and Medicaid Services (CMS) and is based on weekly surveys of pharmacies and their dispensed drugs.^15^ The unit of analysis for our study outcomes is drug-specific measured by NDC code for price outcomes and product name for utilization outcomes. NDCs were used to identify sample drugs in the analysis of price and utilization levels and trends.

### Prices

Prices for branded and generic PrEP were identified using two sources. First, we used the NADAC database, which lists the average prices that pharmacies pay for drugs by NDC code. Second, we used a proprietary database from SSR Health that estimated net prices, i.e., prices after rebates, for branded PrEP by NDC code.^16^ The reason for using a second database for price estimates of branded PrEP is that branded drugs often have manufacturer rebates which may lower their effective drug prices for payers; these rebates are not normally observed amongst generics.^17^ Note that we smoothed the estimated quarterly net prices using a four-quarter moving average since the estimated net prices had quarter to quarter fluctuations, which we believe was an artifact of SSR’s methods to estimate net prices. All prices are presented in a common dosage and form and are per-pill prices.

### National Utilization

National utilization data relevant to branded and generic PrEP was sourced using IQVIA’s National Prescription Audit^®^ (NPA) database, which has monthly dispensing counts from 92% of U.S. retail pharmacies and 70% of U.S. long-term care and mail-order pharmacies.^18^ The database provides estimated number of units sold measured by pills in the U.S. by month for each molecular product identified by product name, and dispensing counts are projected to national totals using IQVIA’s proprietary methodology. Data was presented quarterly. The data extract used for this study did not include the medical indication. The combination drug of emtricitabine and tenofovir disoproxil fumarate and the combination drug of emtricitabine and tenofovir alafenamide fumarate both can be used for two purposes: antiretroviral therapy (ART) for HIV treatment as well as PrEP for HIV prevention. Our extract could not distinguish which dispensed pills were used for which indication (ART or PrEP).

### State Medicaid Utilization

State Medicaid utilization of branded and generic PrEP was sourced using publicly available data from CMS.^19^ These data were collected by CMS as part of the Medicaid Drug Rebate Program (MDRP) and excluded drugs dispensed in the 340B Drug Pricing Program. Data includes drugs used in both fee-for-service and managed care Medicaid programs. Drugs were identified using NDCs and product names. This database also does not distinguish between medical indications of ART or PrEP.

### Statistical Analysis

We conducted exploratory analysis to analyze quarterly levels trends in prices and utilization. This included a visual analysis of the outcomes. We also estimated average annual changes in utilization and prices using data from first quarter 2019 and first quarter 2021 only. We reasoned that this approach avoided the disruptions to the U.S., pharmaceutical market that occurred in 2020 due to the COVID-19 pandemic.^20^

Our study was exempt from Johns Hopkins Bloomberg School of Public Health institutional review board approval because it did not constitute human participants’ research. This study followed the Strengthening the Reporting of Observational Studies in Epidemiology (STROBE) reporting guideline for cross sectional studies.^21^

## Results

**Figure 1:**
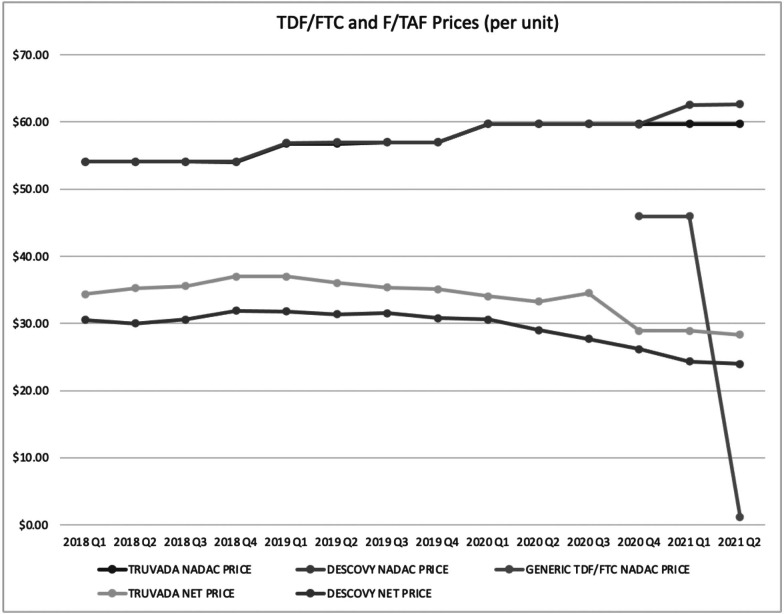
TDF/FTC and F/TAF Prices Q1 2019 to Q2 2021

After two quarters of exclusivity for generic PrEP, prices dropped substantially to approximately one dollar per dose compared to an estimated net price of $28 per dose for branded PrEP (see Figure 1). For branded PrEP, we saw a small price drop of 5% for Descovy from third quarter 2020 to fourth quarter 2020, corresponding to when generic PrEP entered the U.S. market. We saw a larger price drop (17%) for Truvada during the same time period. In first quarter 2021, generic PrEP represented 28% of total prescription volumes for PrEP in the U.S (see Table 1). As of first quarter 2021, there were nine generic manufacturers of generic PrEP.

**Table 1:** Prescription Volumes of TDF/FTC and F/TAF

	**Total U.S. Market** **(Prescription volume)**				**Total Medicaid Market** **(Prescription volume)**			
Quarter	Truvada	Descovy	Generic TDF/FTC	Generic Uptake	Truvada	Descovy	Generic TDF/FTC	Generic Uptake
2019 Q1	446,596	189,684			68,819	57,369		
2019 Q2	469,681	183,882			65,643	53,045		
2019 Q3	468,233	179,988			67,877	52,546		
2019 Q4	416,364	241,172			58,254	51,927		
2020 Q1	344,859	313,898			52,143	54,548		
2020 Q2	287,933	334,566			46,115	55,660		
2020 Q3	281,662	352,453			47,118	58,356		
2020 Q4	149,500	352,773	123,954	19.8%	24,903	57,427	19,455	19.1%
2021 Q1	102,049	345,766	177,632	28.4%	17,922	52,824	23,206	24.7%

**Figure 2:**
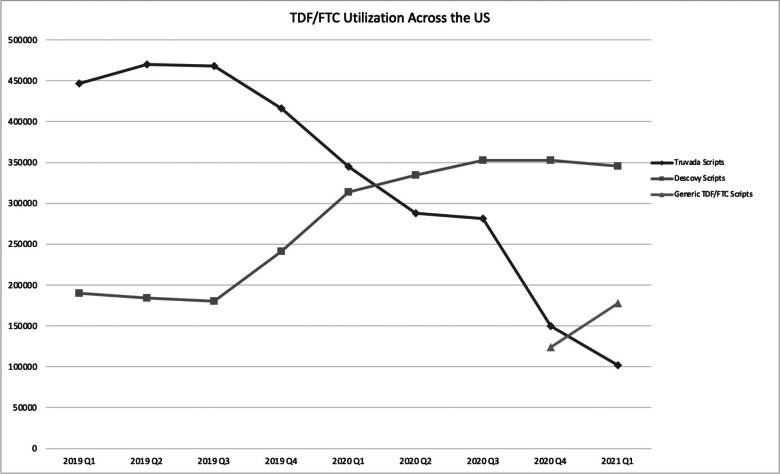
TDF/FTC and F/TAF Prescription Volumes over Time (U.S. Market)

**Figure 3:**
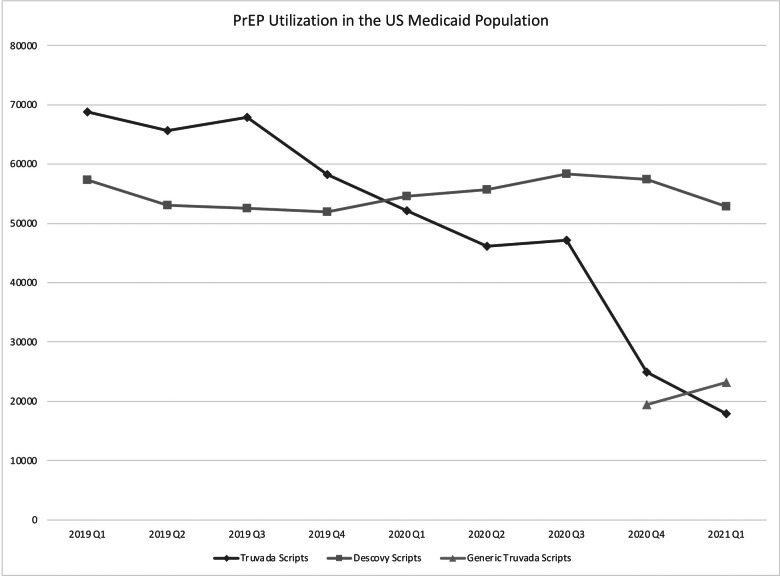
PrEP Prescription Volumes over Time (Medicaid)

Despite the lower price, changes in total prescription volume across the U.S. for all PrEP drugs decreased by approximately 1% between first quarter 2019 and first quarter 2021 (see Figure 2). When examining prescription volume changes strictly for branded PrEP, we observed a drop of approximately 30% between first quarter 2019 and first quarter 2021. When examining state Medicaid program’s utilization of generic PrEP, we observed generic PrEP has a 25% penetration rate based on prescription volumes (see Table 1). When comparing Medicaid volumes of PrEP between first quarter 2019 and first quarter 2021, we find an approximate 25% decrease in PrEP volumes (see Figure 3).

## Discussion

### Opportunities Presented by the Availability of Generic PrEP

In the PrEP market, we observed a 97% price drop for generic versions compared to branded estimated net prices in the first half of 2021. This result is consistent with previous studies suggesting drug prices decrease significantly with loss of exclusivity, generic entry and competition.^22^ The lower price of generic PrEP observed holds promise for reducing observed access challenges to PrEP, and it may help the U.S. to reach its 2030 goal of increasing PrEP uptake by 50%.^23^ By lowering drug prices, generic drugs increase affordability for patients and may help efforts to improve access to crucial medications among HIV vulnerable populations.^24^

Nonetheless, we observed low penetration of generic PrEP and little evidence of PrEP access expansion after generic drug entry and competition. In fact, from 2019 to 2021, we found a 1% decrease in total PrEP prescription volume. The decrease in total prescription volume of PrEP is disconcerting and is even more worrisome given that the U.S. Prevention and Screening Task Force gave PrEP an “A” rating in 2019.^25^ The “A” rating means most private health plans must cover this medication with no form of patient cost sharing, and with low generic prices for payers and no cost-sharing, one would have expected an increase in PrEP uptake. Examining the Medicaid market, our finding of a 25% reduction in Medicaid PrEP dispensing may reflect Medicaid populations gaining access to PrEP through other channels outside of Medicaid programs.

The question then becomes why are we not seeing an overall increase in prescription volumes of PrEP given the availability of lower priced generics? There are several possible reasons. First, generic PrEP was only FDA approved in October 2020 and our utilization data only accounts for the first three quarters after approval. It is worth noting that generic PrEP prices did not fall drastically until the second quarter 2021 since the sponsor, Teva Pharmaceuticals, was the only manufacturer with a generic on the market at the time and thus was able to maintain relatively high prices due to lack of generic competition. The lack of competition was a result of a settlement between Gilead and Teva where Gilead let Teva be the sole manufacturer of generic PrEP for six months.

Another possible explanation for the stagnant total prescription volumes of PrEP may be due to the need of support services to utilize PrEP. Individuals wishing to use PrEP need provider visits to establish care, laboratory tests, and significant follow-up to ensure PrEP is being taken appropriately. The availability of lower priced generic PrEP, i.e., does not automatically improve access or affordability of these important complementary support services.

A third explanation is that the COVID-19 pandemic disrupted access to and demand for PrEP. The historic volume of job loss at the beginning of the pandemic resulted in disruptions in insurance coverage which might have discouraged continuing medical care.^26^ In addition, pandemic-associated social distancing measures were shown to change sexual behaviors of gay men, who reported being less likely to have casual sex or feel the need to continue using PrEP.^27^ Finally, the U.S. prescription drug market includes some perverse incentives for providers to prescribe and pharmacies to dispense name-brand drugs even when lower prices generics are available we discuss in detail below.

### Role of 340B

For many low-income and uninsured patients, the 340B drug discount program facilitates access to prescription drugs. The program allows eligible, largely safety-net clinics, hospitals and pharmacies, to acquire prescription drugs at deep discounts,^28^ estimated to be 20-50% off regular purchase prices. Discounted prices may be passed onto patients in the form of lower out of pocket costs, and consequently increasing medication affordability at the pharmacy counter.

However, 340B discounts can also act as an impediment to generic drug uptake. 340B clinics, hospitals, and pharmacies are not required to pass the discounts to patients, but rather discounts can be retained by charging patients and payers full prices and thus generating revenue for the provider off the difference.^29^ This is colloquially known as “ spread pricing.” Spread pricing is a way for the 340B program to meet its congressional intent which is to “stretch scarce Federal resources as far as possible, reaching more eligible patients and providing more comprehensive services.”^30^ However, the program does not specify how this money is to be reinvested, nor are there any reporting mechanisms in the policy to ensure it goes towards Congress’s stated goals. Furthermore, the evidence around 340B entities reinvesting profit obtained through spread pricing to improve access is mixed.^31^ In the context of drugs with brand and generic versions, providers face incentives to use the higher priced branded drug since the 340B discount is calculated as a percentage of the drug’s price.

The 340B drug discount program has an outsized influence on the delivery of HIV medications in the U.S. — previous work has found that antiretroviral medications for HIV account for the top three most common 340B prescriptions.^32^ In 2021, Killelea and Horn outlined and discussed the challenges for generic uptake of PrEP and the 340B program.^33^ They identified the perverse incentive for 340B providers to administer the more expensive Descovy, despite little clinical evidence of superior health benefits to branded or generic Truvada.^34^

For federally qualified health centers (FQHCs), the extra revenue from selling higher cost branded drugs when generics are available is less likely a windfall and more likely a substantial portion of the budget for these providers, which are statutorily required to provide care on a sliding scale based on a patient’s ability to pay.^35^ FQHCs reduce cost-related access barriers and provide culturally-competent care that is tailored to patient populations disproportionately burdened by HIV, including LGBTQ individuals. In January 2022, Gilead ended its practice or reimbursing 340B providers dispensing PrEP for patients on Gilead’s Advancing Access patient assistance program at a usual and customary price. By reimbursing 340B providers at acquisition cost for these patients, Gilead has eliminated the spread 340B providers were able to generate from the Advancing Access program for uninsured patients.^36^ These dynamics complicate generic PrEP’s ability to reduce access barriers through reduced cost.

Policymakers have an opportunity to leverage the availability of lower priced generic PrEP to help achieve the 2030 goal of 50% PrEP uptake in HIV vulnerable individuals. To do this, policymakers should entertain a national strategy approach to minimize geographic variation. Lower priced generic PrEP can facilitate direct federal purchasing without a major budgetary burden. Direct federal purchasing of PrEP should be coupled with federal financing of laboratory services to ensure patients have access to the drug and necessary support of care. The target population of a federal policy should be the uninsured and Medicaid populations who are currently under utilizers of PrEP and are reliant on several disjointed schemes for access.

### Policy Solutions

Policymakers have an opportunity to leverage the availability of lower priced generic PrEP to help achieve the 2030 goal of 50% PrEP uptake in HIV vulnerable individuals. To do this, policymakers should entertain a national strategy approach to minimize geographic variation. Lower priced generic PrEP can facilitate direct federal purchasing without a major budgetary burden. Direct federal purchasing of PrEP should be coupled with federal financing of laboratory services to ensure patients have access to the drug and necessary support of care. The target population of a federal policy should be the uninsured and Medicaid populations who are currently under utilizers of PrEP and are reliant on several disjointed schemes for access. Killelea and colleagues present a detailed proposal along these lines.^37^

### Limitations

This study has limitations. First, the combination drug of emtricitabine and tenofovir disoproxil fumarate and the combination drug of emtricitabine and tenofovir alafenamide fumarate are not exclusively used for PrEP since they are also used for HIV management. Since the IQVIA NPA and Medicaid data lack clinical details, we are unable to determine what proportion of quarterly national dispensing is indicated for PrEP and what is indicated for HIV management. Second, our study only examines prescription volume up to first quarter 2021. Generic PrEP was only on the market for two quarters, and it may be too early to generalize on generic PrEP uptake.

## Conclusions

In this analysis, the introduction of generic PrEP significantly reduced the per-unit price of the drug. Lower priced generic PrEP should result in increased overall uptake of PrEP; however, our analysis of early utilization patterns of generic PrEP suggests generic PrEP may be cannibalizing branded PrEP. While generic PrEP holds promise to reduce cost-related access barriers and help the U.S. reach the goal of 50% uptake of PrEP in the US by 2030, structural challenges in the PrEP market such as the 340B provider incentives may hinder increased PrEP uptake.

## Note

Arnold Ventures (JB, RMC). American Cancer Society (RMC). Timothy W. Levengood was supported by the National Institute on Drug Abuse of the National Institutes of Health under award number T32DA04189803. The study sponsor had no role in study design; collection, analysis, and interpretation of data; writing the report; and the decision to submit the report for publication. Ballreich has reported a grant from the US Food and Drug Administration.
